# Supplementary material for: Synthetic fascia for stiff and tough 4D printed multifunctional structures that detect and tolerate damage
Source: Nat Commun. 2025 Nov 24;16:10351. doi: 10.1038/s41467-025-65279-w (PMC12644615; doi:10.1038/s41467-025-65279-w)
Supplement: Supplementary file 1 — Supplementary Information [file 41467_2025_65279_MOESM1_ESM.pdf]

Synthetic fascia for stiff and tough 4D printed multifunctional structures that detect and tolerate damage

Javier M Morales Ferrer[1], Chloe Kekedjian[2], Nicole Bacca[1], and J. William Boley[1,2]*

1. Boston University Department of Mechanical Engineering
   110 Cummington Mall, Boston, MA 02215, USA
2. Boston University Division of Materials Science and Engineering
   15 St Mary's St, Boston, MA 02215, USA

* Email Address: jwboley@bu.edu

Supplementary Section

Methods

Modeling Optimal Volume Fraction for Synthetic Muscle Composite

We implemented the rule of mixture to model the effective elastic modulus of the synthetic muscle composite, composed of low α MHEC ink and synthetic fascia (MS 1:1) ink. Both materials are considered to be homogeneous and elastically transversely isotropic along the printed direction [1]. The expression for elastic modulus of the composite along the printed direction is given by,

$$E_c = E_{MHEC} f_{MHEC} + E_{SF} f_{SF}, \tag{1}$$

where E_{MHEC} and E_{SF} are 33.43 GPA and 2.48 MPa, respectively. The results for this model are shown with a dotted line in Figure 2c.

Electro-Thermo-Mechanical Modeling Details

Modeling of the responsive bilayers is built upon findings further explained in previous work [2]. Here we slightly edited the model to compensate for the additional PDMS layer. Essentially the main difference is contained in the thermal properties of the composite (Specific heat at constant volume (C_v) and density (ρ)), and the heating volume. To account for these changes, we integrated the rule of mixture [3] to determine the ρ and C_v for the synthetic muscle composite, denoted as ρ_c and C_{v_c}, respectively. The expression use for $\rho_c = \rho_{MHEC} f_{MHEC} + \rho_{SF} f_{SF}$ ($\rho_{MHEC} \sim 1333.2kgm^{-3}, \rho_{SF} \sim 1000kgm^{-3}$) and $C_{v_c} = C_{v_{MHEC}} f_{MHEC} + C_{v_{SF}} f_{SF}$ ($C_{v_{MHEC}} \sim 1110Jkg^{-1}C^{-1}$, $C_{v_{SF}} \sim 1460Jkg^{-1}C^{-1}$), where f is the volume fraction of the constituent in the composite. The heating volume for the composite know is described as $V_c \sim w_c L_c h_c$, where w, L, and h represent width, length, and height, respectively. Mainly, these changes affected the thermal responses time which is described by the time constant expression as

$$\tau = \frac{\rho_c C_{v_c} V_c}{B}, \tag{2}$$

$$B = h_\perp A_\perp + 2h_\parallel A_\parallel, \tag{3}$$

Where h, and A represents the convective coefficient and surface area, respectively. The symbols $\perp$ and $\parallel$ represent transverse and longitudinal (respectively), referring to the orientation of the samples with respect to the heat losses. To calculated $A_\perp$ and $A_\parallel$ we assumed a rectangular cross section, hence $A_\perp \sim w_c L_c$ and $A_\parallel \sim h_c w_c$. The convective coefficients are determined by an iterative process, further explained in previous work [2]. Using Equation 2, we calculated the minimum τ to be ~ 20.41 seconds.

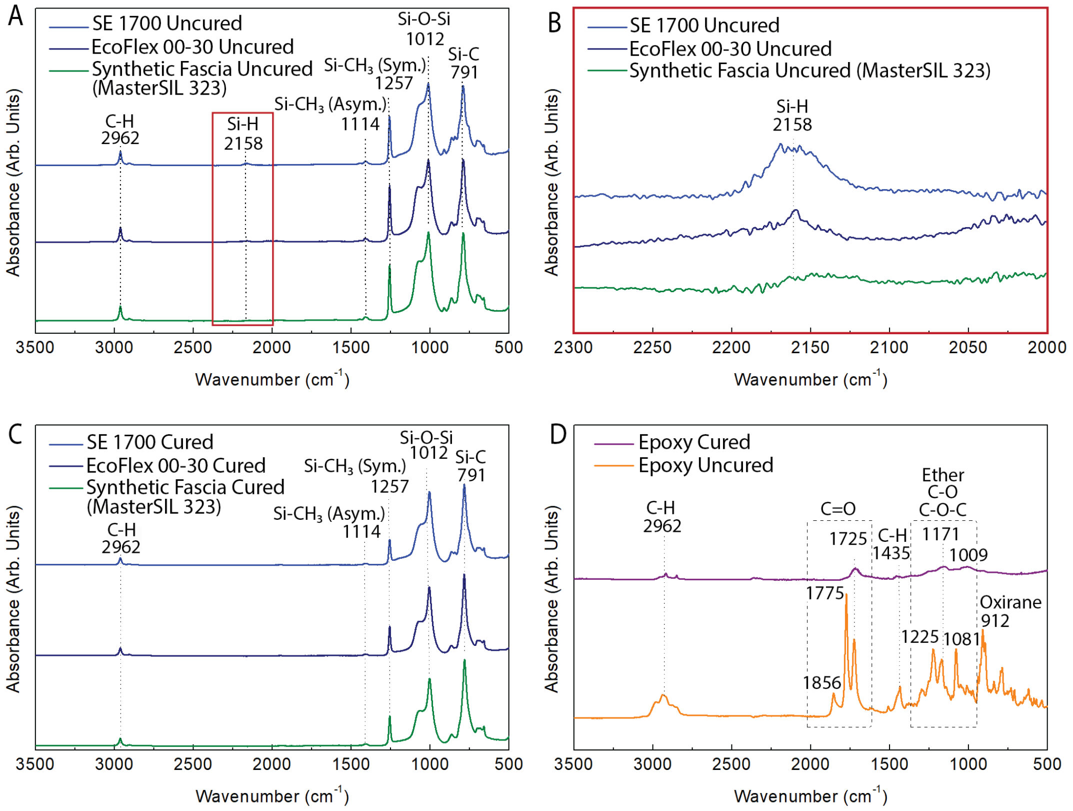

Figure S1: FTIR analysis of PDMS and Epoxy. a) FTIR plot of uncured PDMS (SE1700, EcoFlex 00-30, and MasterSIL 323 (SF)). The FTIR data showed similar characteristic peaks of PDMS for most of the samples including aliphatic C-H stretching at 2962 cm^{-1}, asymmetric and symmetric deformation of Si-CH_3 at 1412 cm^{-1} and 1257 cm^{-1}, long chain and cyclic siloxane Si-O-Si stretching between 1000 - 1100 cm^{-1}, and Si-C stretching at 791 cm^{-1} [4] [5]. While there was some variation in the fingerprint region, the three uncured PDMS samples were relatively similar, other than the height of the 2160 cm^{-1} Si-H peak, which was large for EcoFlex 00-30 and SE1700. b) Expanded view of hydrosilanol peak of PDMS at 2158 cm^{-1} c) FTIR plot of cured PDMS. The cured PDMS shares the same peaks, but the flattening of the peak at 2160 cm^{-1}confirms that curing has occurred. d) FTIR of uncured and cured neat epoxy (Supreme 112SP, Masterbond). The neat uncured epoxy shows aliphatic C-H stretching at 2952 cm^{-1} and asymmetric deformation of Si-CH_3 at 1435 cm^{-1}. There are series of peaks between 1725-1856 cm^{-1}, corresponding to ketone stretches, and 1081-1225 cm^{-1}, corresponding to ether stretches [6]. There is a pronounced oxirane peak at 912 cm^{-1} [7]. The cured epoxy showed some aliphatic C-H stretching, ketone stretching and ether stretching, though those peaks were far less pronounced. Additionally, the oxirane peak was missing from the cured epoxy plot, indicating that curing has occurred.

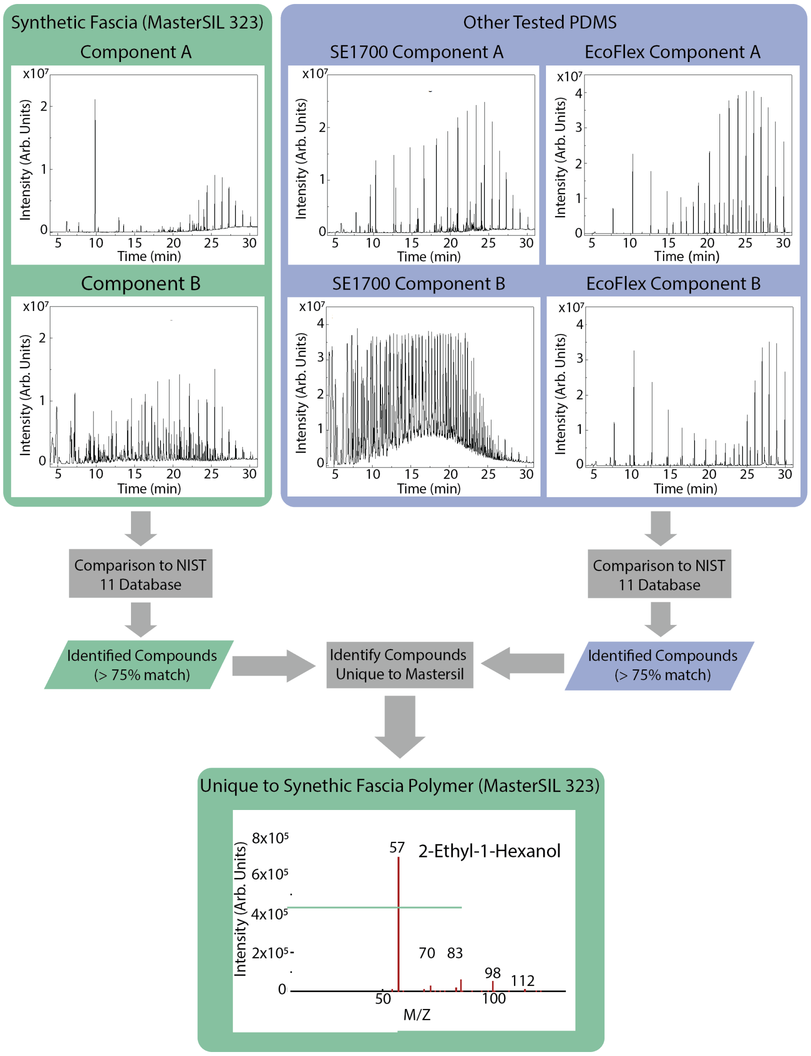

Figure S2: Workflow of Gas Chromatography Mass Spectrometry (GC-MS) of polymer precursors. Full chromatograms of MasterSIL A, MasterSIL B, SE1700 A, SE1700 B, EcoFlex 00-30 A, and EcoFlex 00-30 B, were compared to the National Institute of Standards and Technology Mass Spectral Database (NIST 11). Compounds with a greater than 75 % match were compared between polymers to identify compounds unique to MasterSIL. The mass spectrum of a compound (retention time = 6.147 min, intensity = $1.75 \times 10^7 \pm 0.02 \times 10^7$) matching 2-ethyl-1-hexanol (average match of 79.7 ± 2.9) was identified as unique to MasterSIL A. These polymers contained other compounds that could not be identified via the database, as well as shared cross-linkers and adhesion promoters, which could also contribute to their adhesive properties.

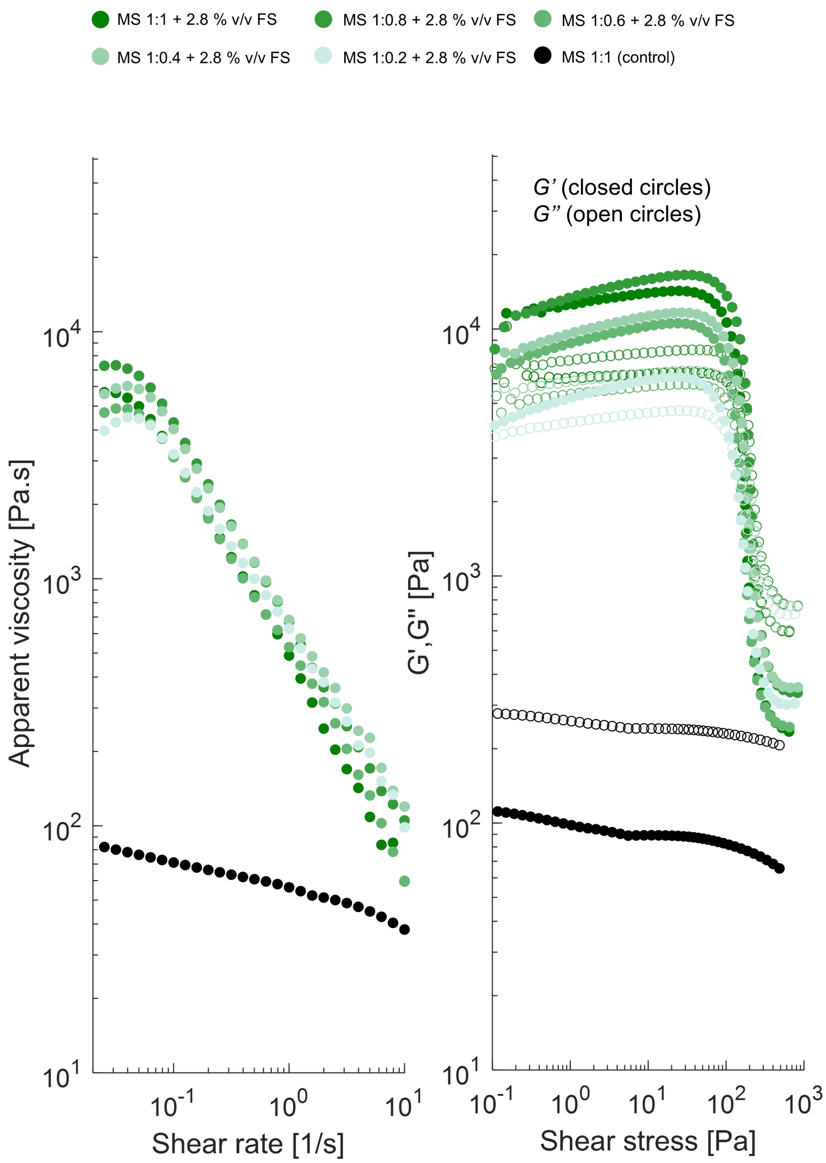

Figure S3: Ink rheology for SF formulations. Log-log plots of the apparent viscosity with respect to the shear rate for printable inks (tones of greens) and neat PDMS resin (black) (left). Log-log plot of the storage (G', closed circle) and loss (G'', open circle) moduli as a function of shear stress for printable inks (tones of greens) and neat PDMS resin (black) (right).

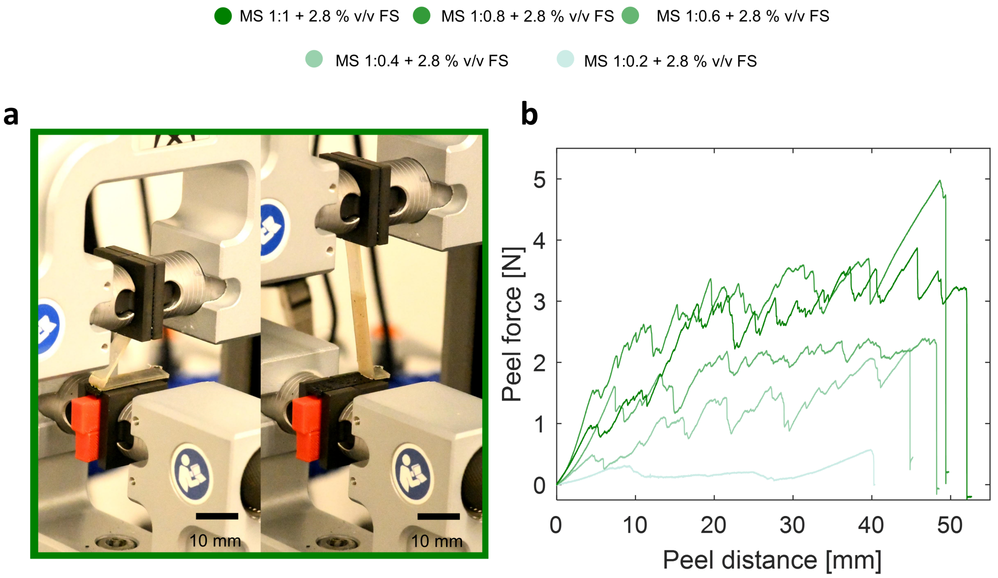

Figure S4: Characterization of adhesion of SF to MHEC. a) Representative photographs of the 90° peeling experimental setup used in this study, sample used for this testing was MS 1:1 + 2.8 % v/v FS. b) A summary of the peel force versus peel length for different test specimens. Different tones of green represent different SF base-to-crosslinker formulations.

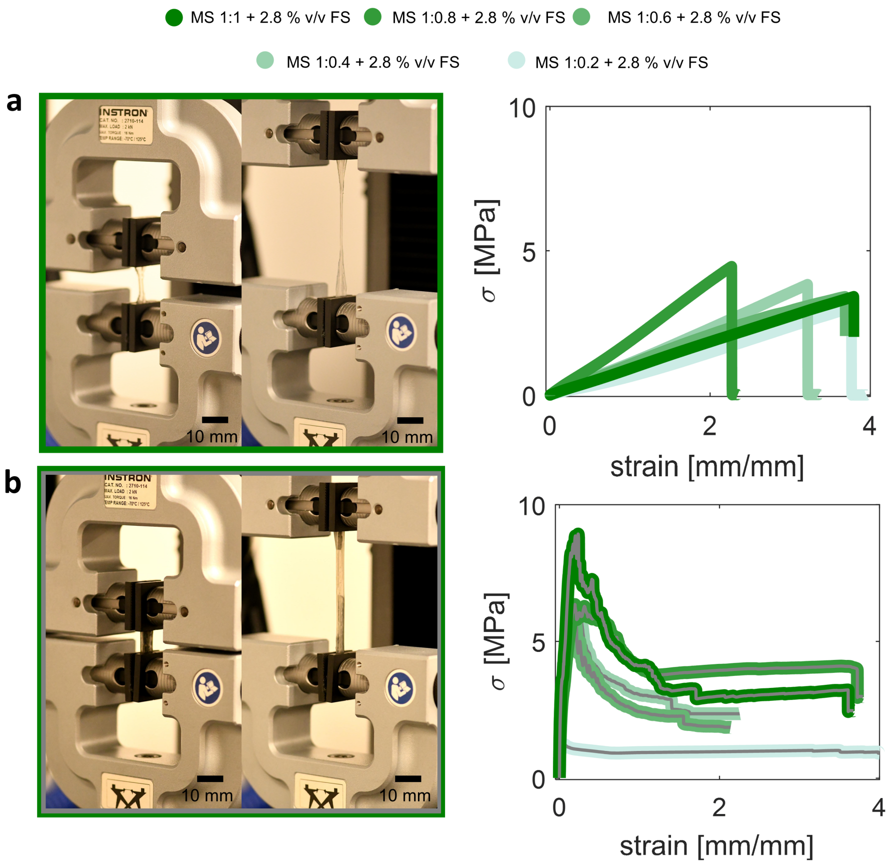

Figure S5: Tensile testing for characterize E and K for various SF formulations. a) Representative photographs of a MS 1:1 + 2.8 % v/v FS sample being tested (left). Stress versus strain results for different SF inks formulations (right). b) Representative photographs of testing a synthetic muscle composite comprise of MS 1:1 + 2.8 % v/v FS and 1:0.2 + 15 % v/v CF + 8 % v/v CB (left). Stress versus strain results for different synthetic muscle composite formulations, swapping the SF formulation while keeping the MHEC fix (right). Different tones of green represent different SF base-to-crosslinker formulations. The gray core in the data represent that sample corresponds to the synthetic muscle composite.

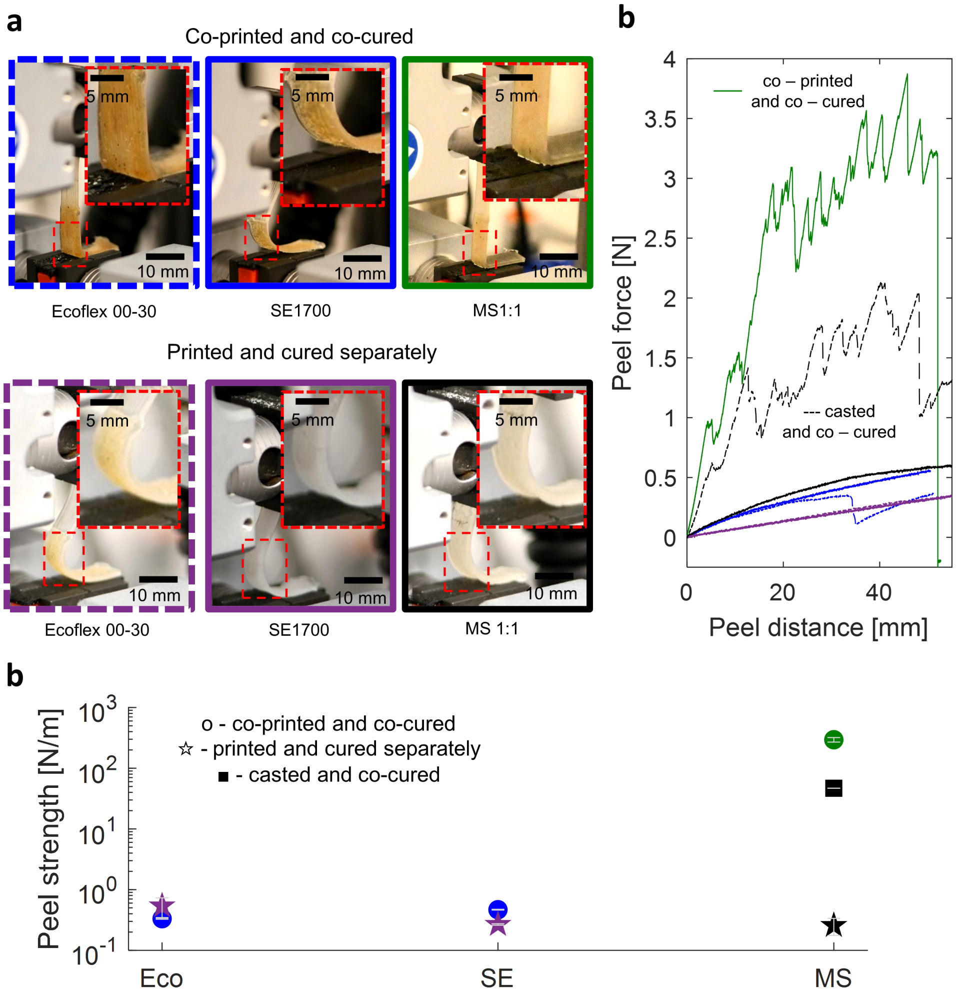

Figure S6: Assessment of different PDMS adherence to MHEC. a) Photographs of different commonly used PDMS being tested for adherence to MHEC using 90° peel off testing. b) A summary of the peel force versus peel length for different test specimens. c) Summary of the peel off strength results with respect to different PDMS, where ECO, SE, and MS stand for Ecoflex 00-30, SE1700, and Master Sil 1:1, respectively. Star and circle markers represent printed and cured separately and co-printed and co-cured, respectively. Square markers represent casted and co-cured samples.

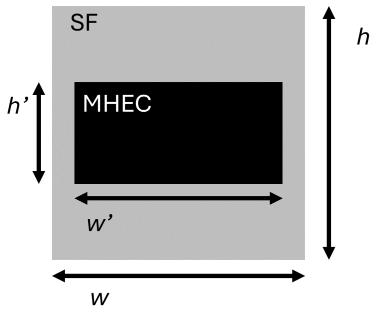

$$v_{MHEC} = (w' x\, h') \, x\, L$$
$$v_{SF} = ((w - w') \cdot h' + (h - h') \cdot w) \cdot L$$
$$v_{Total} = (w\, x\, h)\, x\, L$$

w [mm]	w' [mm]	h [mm]	h' [mm]	f_{SF}	f_{MHEC}
0	1.6	0	1.85	0	1
1.91	1.75	1.85	1.79	0.11	0.89
1.91	1.6	1.85	1.76	0.20	0.80
1.91	1.6	1.85	1.34	0.39	0.61
1.91	1.6	1.85	0.89	0.60	0.40
1.91	1.6	1.85	0.39	0.82	0.18
1.91	0	1.85	0	1	0

$$f_{SF} = \frac{v_{SF}}{v_{SF} + v_{MHEC}} \quad f_{MHEC} = \frac{v_{MHEC}}{v_{SF} + v_{MHEC}}$$

Figure S7: Diagram of volume fraction proportions between MHEC and SF.

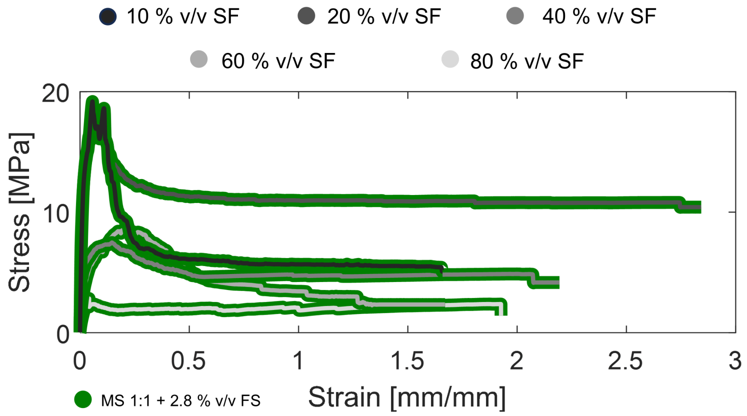

Figure S8: Optimization of E and K for synthetic muscle composite. Summary of stress versus strain results for different synthetic muscle proportions, represented by different grayscale tones. SF and MHEC used in this study are MS 1:1 + 2.8 v/v % and 1:0.2 + 15 % v/v CF + 8 % v/v CB, respectively.

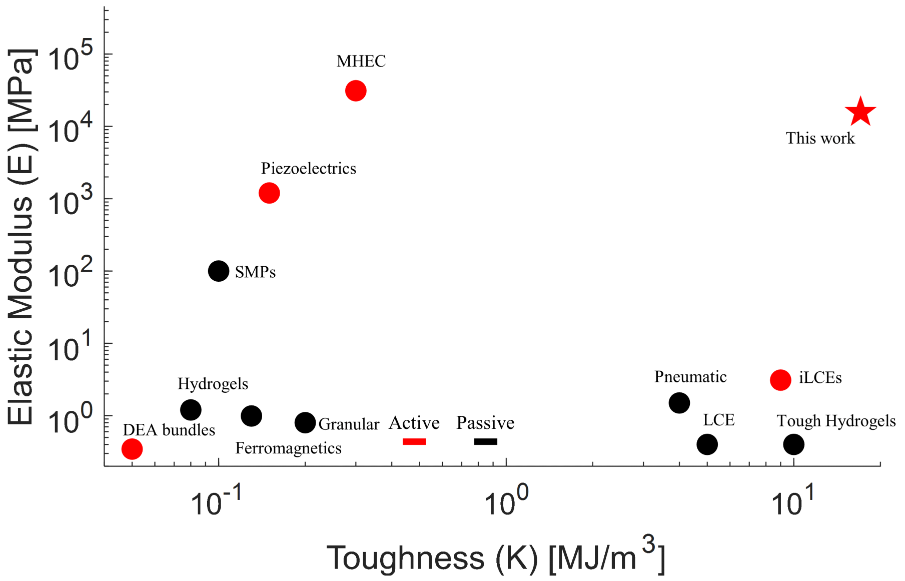

Figure S9: Comparison of E and K of different printable actuators. The red star represents the synthetic muscle composite presented in this work. Black and red data points represent passive and active actuators, respectively. Data for other printable materials is presented in Table S2.

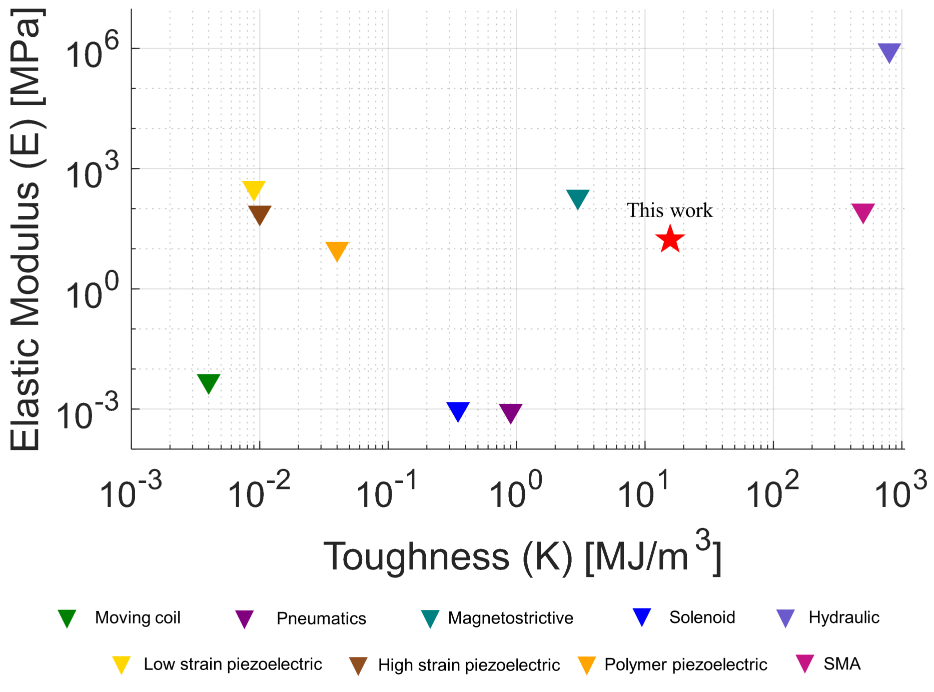

Figure S10: Comparison of E and K of our synthetic muscle composite with commercially available actuators. The red star represents the synthetic muscle composite presented in this work. Data for other printable materials is presented in Table S4.

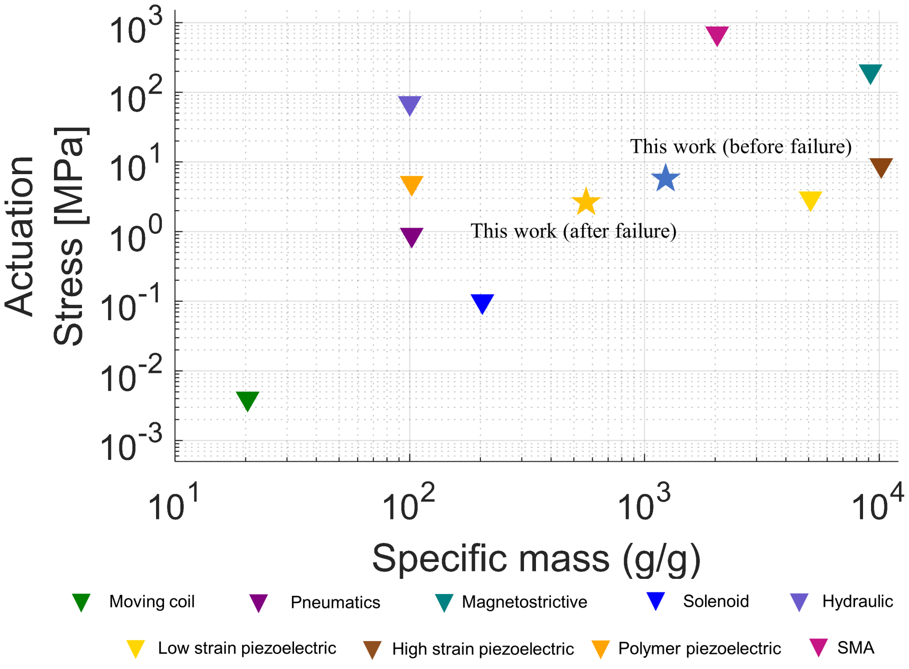

Figure S11: Comparison of our 4D printed actuator with commercially available options, using actuation stress and specific mass as the metrics for comparison. The blue and yellow star represents our active lifting robot before and after failure, respectively. Data for commercially available actuators was extracted from [2].

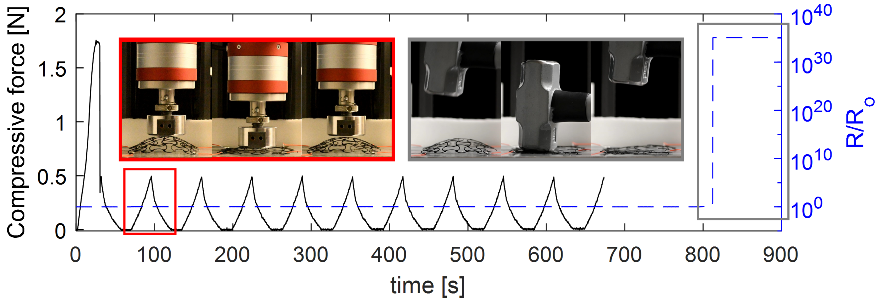

Figure S12: Characterization of MHEC lattice under cycling compressive loads and a high impact load. Red inserts represent the cycling compressive loads exerted on the sample. The gray inserts represented the exerted high impact load on the sample.

Table S1: Summary of elastic modulus and toughness of 4D printable materials while undergoing shape-change.

Material	Elastic modulus (E) during shape change [MPa]	Toughness (K) [MJ m^{-3}]	Ref
Synthetic muscle composite	483.2 - 21,410	4.70 - 27.04	This work
MHECs	337.9 - 36,500	0.01 - 1	[2]
PDMS	0.01 - 1.2	0.13 - 0.5	[8, 9]
Hydrogels	0.02 - 1.3	0.01 - 10	[10, 11, 12]
LCEs	1.5 - 12	2.5 - 9	[13, 14, 15]
SMPs	0.1 - 10	0.1 - 0.2	[16, 17]

Table S2: Mechanical properties of 3D and 4D printable actuators.

Material	Elastic Modulus (E) [MPa]	Toughness (K) [MJ m^{-3}]	Ref
Synthetic muscle composite 4D push mode lifting robot	15,700	17.07	This work
MHEC 4D push mode lifting robot	31,117	0.3	[2]
iLCEs	3.1	$\sim$9	[15]
Polymer piezoelectric	1200	$\sim$0.15	[18]
Ferromagnetic-PDMS	0.99	$\sim$0.13	[9]
SMPs	100	$\sim$0.1	[16]
LCEs	0.4	$\sim$5	[14]
Pneumatic	1.5	$\sim$4	[19]
DEAs bundles	0.345	$\sim$0.05	[20]
Granular gripper	0.8	$\sim$0.2	[21]
Tough Hydrogels	0.5	$\sim$10	[12]
Hydrogels	1.2	$\sim$0.08	[10]

[a] $K_{\text{composite}} = \sum_{i=1}^{n} f_{\text{i}} K_{\text{i}}$, where K_{i} is toughness of material i and f_{i} is volume fraction of material i[22, 23]

[b] $E_{\text{composite}} = \sum_{i=1}^{n} f_{\text{i}} E_{\text{i}}$, where E_{i} is stiffness of material i and f_{i} is volume fraction of material i [22, 23]

Table S3: Mechanical properties of 3D and 4D commercially available actuators.

Material	Elastic Modulus (E) [GPa]	Toughness (K) [MJ m^{-3}]
Shape Memory Alloys (SMAs)	90	500
Hydraulic	3	800
Pneumatic	9 x 10^{-4}	0.9
Magnetostrictor	200	3
Solenoid	1 x 10^{-3}	0.35
High strain piezoelectric	80	0.01
Low strain piezoelectric	330	9 x 10^{-3}
Piezo polymer	10	0.04
Moving coil	5 x 10^{-3}	4 x 10^{-3}

[a] Data extracted from reference [24]

Table S4: Formulations

ID	Base (v/v) [%]	Crosslinker (v/v) [%]	FS (v/v) [%]	Slow Jo (v/v) [%]	CF (v/v) [%]	CB (v/v) [%]	Tr - x (v/v) [%]
MHEC electrically conductive	64.95	12.23	—	—	14.17	8.66	—
MHEC electrically insulative	38.74	29.49	7.85	—	—	—	23.92
Ecoflex 00-30[a]	41.72	52.15	5.03	1.10	—	—	—
SE1700[b]	88.20	8.82	2.97	—	—	—	—
MS 1:0.2	80.99	16.20	2.81	—	—	—	—
MS 1:0.4	69.42	27.77	2.81	—	—	—	—
MS 1:0.6	60.74	36.45	2.81	—	—	—	—
MS 1:0.8	53.99	43.20	2.81	—	—	—	—
MS 1:1	48.60	48.60	2.81	—	—	—	—

[a] Formulation adopted from [25]. [b] Formulation adopted from [8, 26].

Table S5: Printing parameters.

ID	Speed [mm/s]	Pressure [psi]	Nozzle size [mm]	Initial print height [mm]
MHEC electrically conductive	42.5	11	0.410	0.369
MHEC electrically insulative	15	48	0.410	0.369
Ecoflex 00-30	40	12	0.410	0.369
SE1700	40	16	0.410	0.369
MS 1:0.2	40	11	0.410	0.369
MS 1:0.4	40	11	0.410	0.369
MS 1:0.6	40	11.5	0.410	0.369
MS 1:0.8	40	12	0.410	0.369
MS 1:1	40	13	0.410	0.369

* Pressure reported here is the controller pressure, the pressure exerted in the material is the reported pressure times 7x

Table S6: Stiffness and toughness summary of formulations.

ID	Stiffness (E) [MPa]	Standard deviation [MPa]	Toughness (K) [MJ/m^3]	Standard deviation [MJ/m^3]
MHEC electrically conductive	31,430.00	2,750.00	0.31	0.02
MHEC electrically insulative	10,460.00	820.00	0.27	0.04
Ecoflex 00-30	0.125	0.34	1.6	0.28
SE1700	1.25	0.22	2.01	0.23
MS 1:0.2	0.60	0.06	4.29	0.55
MS 1:0.4	1.63	0.27	5.64	0.39
MS 1:0.6	1.95	0.18	7.48	0.36
MS 1:0.8	2.15	0.26	15.35	0.40
MS 1:1	2.48	0.38	14.89	0.12
SF(MS 1:1)/MHEC electrically conductive (10 v/v SF)	21,410.01	3,855.12	8.58	0.50
SF(MS 1:1)/MHEC electrically conductive (20 v/v SF)	17,900.12	240.81	27.07	3.91
SF(MS 1:1)/MHEC electrically conductive (40 v/v SF)	6,440.19	1,246.45	14.42	4.78
SF(MS 1:1)/MHEC electrically conductive (60 v/v SF)	2,501.22	285.98	6.32	1.24
SF(MS 1:1)/MHEC electrically conductive (80 v/v SF)	483.21	24.89	4.70	1.01

* Pressure reported here is the controller pressure, the pressure exerted in the material is the reported pressure times 7x

References

[1] R. C. Hibbeler. *Mechanics of Materials.* 2017.

[2] Javier M Morales Ferrer, Ramón E Sánchez Cruz, Sophie Caplan, Wim M van Rees, and J William Boley. Multiscale heterogeneous polymer composites for high stiffness 4d printed electrically controllable multifunctional structures (adv. mater. 8/2024). *Advanced Materials*, 36(8):2470061, 2024.

[3] Theodore L. Bergman, Theodore L. Bergman, Frank P. Incropera, David P. Dewitt, and Adrienne S. Lavine. *Fundamentals of heat and mass transfer.* 2011.

[4] Albert Lee Smith. *Analysis of Silicones , ed. John Wiley & Sons, New York-London-Sydney-Toronto, 1974. 407 pp.* 1974.

[5] Cédric Schneider, Michel Sablier, and Bernard Desmazières. Characterization by mass spectrometry of an unknown polysiloxane sample used under uncontrolled medical conditions for cosmetic surgery. *Rapid Communications in Mass Spectrometry*, 22(21):3353–3361, 2008-09.

[6] Samuel Meure, Dong-Yang Wu, and Scott A. Furman. FTIR study of bonding between a thermoplastic healing agent and a mendable epoxy resin. *Vibrational Spectroscopy*, 52(1):10–15, 2010.

[7] N Poisson, G Lachenal, and H Sautereau. Near-and mid-infrared spectroscopy studies of an epoxy reactive system. *Vibrational spectroscopy*, 12(2):237–247, 1996.

[8] J. William Boley, Wim M. van Rees, Charles Lissandrello, Mark N. Horenstein, Ryan L. Truby, Arda Kotikian, Jennifer A. Lewis, and L. Mahadevan. Shape-shifting structured lattices via multimaterial 4d printing. *Proceedings of the National Academy of Sciences*, 116(42):20856–20862, 2019.

[9] Yoonho Kim, Hyunwoo Yuk, Ruike Zhao, Shawn A. Chester, and Xuanhe Zhao. Printing ferromagnetic domains for untethered fast-transforming soft materials. *Nature*, 558(7709):274–279, 2018.

[10] A. Sydney Gladman, Elisabetta A. Matsumoto, Ralph G. Nuzzo, Lakshminarayanan Mahadevan, and Jennifer A. Lewis. Biomimetic 4d printing. *Nature materials*, 15(4):413–418, 2016.

[11] Skylar Tibbits. 4d printing: multi-material shape change. *Architectural Design*, 84(1):116–121, 2014.

[12] Mutian Hua, Dong Wu, Shuwang Wu, Yanfei Ma, Yousif Alsaid, and Ximin He. 4d printable tough and thermoresponsive hydrogels. *ACS Applied Materials & Interfaces*, 13(11):12689–12697, 2021-03-24.

[13] Arda Kotikian, Ryan L. Truby, John William Boley, Timothy J. White, and Jennifer A. Lewis. 3d printing of liquid crystal elastomeric actuators with spatially programed nematic order. *Advanced materials*, 30(10):1706164, 2018.

[14] Shuo Li, Hedan Bai, Zheng Liu, Xinyue Zhang, Chuqi Huang, Lennard W. Wiesner, Meredith Silberstein, and Robert F. Shepherd. Digital light processing of liquid crystal elastomers for self-sensing artificial muscles. *Science Advances*, 7(30):eabg3677, 2021.

[15] Arda Kotikian, Javier M. Morales, Aric Lu, Jochen Mueller, Zoey S. Davidson, J. William Boley, and Jennifer A. Lewis. Innervated, self-sensing liquid crystal elastomer actuators with closed loop control. *Advanced Materials*, page 2101814, 2021.

[16] Qi Ge, Amir Hosein Sakhaei, Howon Lee, Conner K. Dunn, Nicholas X. Fang, and Martin L. Dunn. Multimaterial 4d printing with tailorable shape memory polymers. *Scientific reports*, 6(1):1–11, 2016.

[17] Changdeng Liu, Haihu Qin, and P. T. Mather. Review of progress in shape-memory polymers. *Journal of materials chemistry*, 17(16):1543–1558, 2007.

[18] Huachen Cui, Desheng Yao, Ryan Hensleigh, Haotian Lu, Ariel Calderon, Zhenpeng Xu, Sheyda Davaria, Zhen Wang, Patrick Mercier, and Pablo Tarazaga. Design and printing of proprioceptive three-dimensional architected robotic metamaterials. *Science*, 376(6599):1287–1293, 2022.

[19] Manuel Schaffner, Jakob A. Faber, Lucas Pianegonda, Patrick A. Rühs, Fergal Coulter, and André R. Studart. 3d printing of robotic soft actuators with programmable bioinspired architectures. *Nature communications*, 9(1):878, 2018.

[20] Alex Chortos, Ehsan Hajiesmaili, Javier Morales, David R. Clarke, and Jennifer A. Lewis. 3d printing of interdigitated dielectric elastomer actuators. *Advanced Functional Materials*, 30(1):1907375, 2020.

[21] Sophia Eristoff, Sang Yup Kim, Lina Sanchez-Botero, Trevor Buckner, Osman Doğan Yirmibeşoğlu, and Rebecca Kramer-Bottiglio. Soft actuators made of discrete grains. *Advanced Materials*, 34(16):2109617, 2022.

[22] Lewis M Cox, Adrienne K Blevins, Jasper A Drisko, Yuan Qi, Yifu Ding, Callie I Fiedler-Higgins, Rong Long, Christopher N Bowman, and Jason P Killgore. Tunable mechanical anisotropy, crack guiding, and toughness enhancement in two-stage reactive polymer networks. *Advanced Engineering Materials*, 21(8):1900578, 2019.

[23] LRF Rose. Effective fracture toughness of microcracked materials. *Journal of the American ceramic society*, 69(3):212–214, 1986.

[24] J. E. Huber, N. A. Fleck, and M. F. Ashby. The selection of mechanical actuators based on performance indices. *Proceedings of the Royal Society of London. Series A: Mathematical, physical and engineering sciences*, 453(1965):2185–2205, 1997.

[25] Ramón E. Sánchez Cruz, Stephanie F. Zopf, and J. William Boley. A 3d printed liquid metal emulsion for low stress activated stretchable electronics. *Journal of Composite Materials*, page 00219983221149255, 2023.

[26] Zhenwei Wang, Congcong Luan, Yuanbo Zhu, Guangxin Liao, Jiapeng Liu, Xiaojuan Li, Xinhua Yao, and Jianzhong Fu. Integrated and shape-adaptable multifunctional flexible triboelectric nanogenerators using coaxial direct ink writing 3d printing. *Nano Energy*, 90:106534, 2021.
