# Supplementary material for: Synthetic fascia for stiff and tough 4D printed multifunctional structures that detect and tolerate damage
Source: Nat Commun. 2025 Nov 24;16:10351. doi: 10.1038/s41467-025-65279-w (PMC12644615; doi:10.1038/s41467-025-65279-w)
Supplement: Supplementary file 2 — Description of Additional Supplementary Files [file 41467_2025_65279_MOESM2_ESM.pdf]

Description of Additional Supplementary Files

Movie 1: Fabrication of the composite bilayer via multi-material 4D printing. Three materials are printed to produce this structure: synthetic fascia (Green), high α MHEC (Blue), and low α MHEC (Red). Each ink is dyed with a different fluorophore to aid in visualizing the printing process.

Movie 2: Optical characterization of electrically controllable synthetic muscle composite bilayers being actuated via Joule heating with a power of 6.57 W. The testing is carried out for a sample with no fractures (top) and seven fractures (bottom), respectively.

Movie 3: Thermal imaging of a 4D printed synthetic muscle composite bilayers being actuated via Joule heating with a power of 2.84 W. This sample had three induces fracture along the length of the arc.

Movie 4: Fabrication of the composite lifting robot via multi-material 4D printing. Three materials are printed to produce this structure: synthetic fascia (Green), high α MHEC (Blue), and low α MHEC (Red). Each ink is dyed with a different fluorophore to aid in visualizing the printing process.

Movie 5 : Testing of the push-mode stiff and tough lifting robot performance with respect to different external weights, before mechanical failure.

Movie 6 : Testing of the push-mode stiff and tough lifting robot performance with respect to different external weights, after mechanical failure.

Movie 7: Fabrication of the composite lattice via multi-material 4D printing. Three materials are printed to produce this structure: synthetic fascia (Green), high α MHEC (Blue), and low α MHEC (Red). Each ink is dyed with a different fluorophore to aid in visualizing the printing process.

Movie 8: Testing of the MHEC lattice with no SF encapsulation layer.

Movie 9: Testing of the stiff and tough electrically responsive lattice with SF encapsulation layer.

Movie 10: Driving over the stiff and tough electrically responsive lattice with SF encapsulation layer.

Movie 11: Testing actuation of the stiff and tough electrically responsive lattice with SF encapsulation layer, after being driven with a car.

Movie 12: Fabrication of a peel testing sample via multi-material 4D printing. Three materials are printed to produce this structure: synthetic fascia (Green), high α MHEC

(Blue), and low α MHEC (Red). Each ink is dyed with a different fluorophore to aid in visualizing the printing process.

Movie 13: Fabrication of a toughness sample via multi-material 4D printing. Three materials are printed to produce this structure: synthetic fascia (Green), high α MHEC (Blue), and low α MHEC (Red). Each ink is dyed with a different fluorophore to aid in visualizing the printing process.
